# Supplementary material for: Mechanism of cis-Nerolidol-Induced Bladder Carcinoma Cell Death
Source: Cancers (Basel). 2023 Feb 3;15(3):981. doi: 10.3390/cancers15030981 (PMC9913136; doi:10.3390/cancers15030981)
Supplement: Supplementary file 1 [file cancers-15-00981-s001.zip › File S1.pdf]

Mechanism of *Cis*-Nerolidol-Induced Bladder Carcinoma Cell Death

Raw blots

Figure S1 C

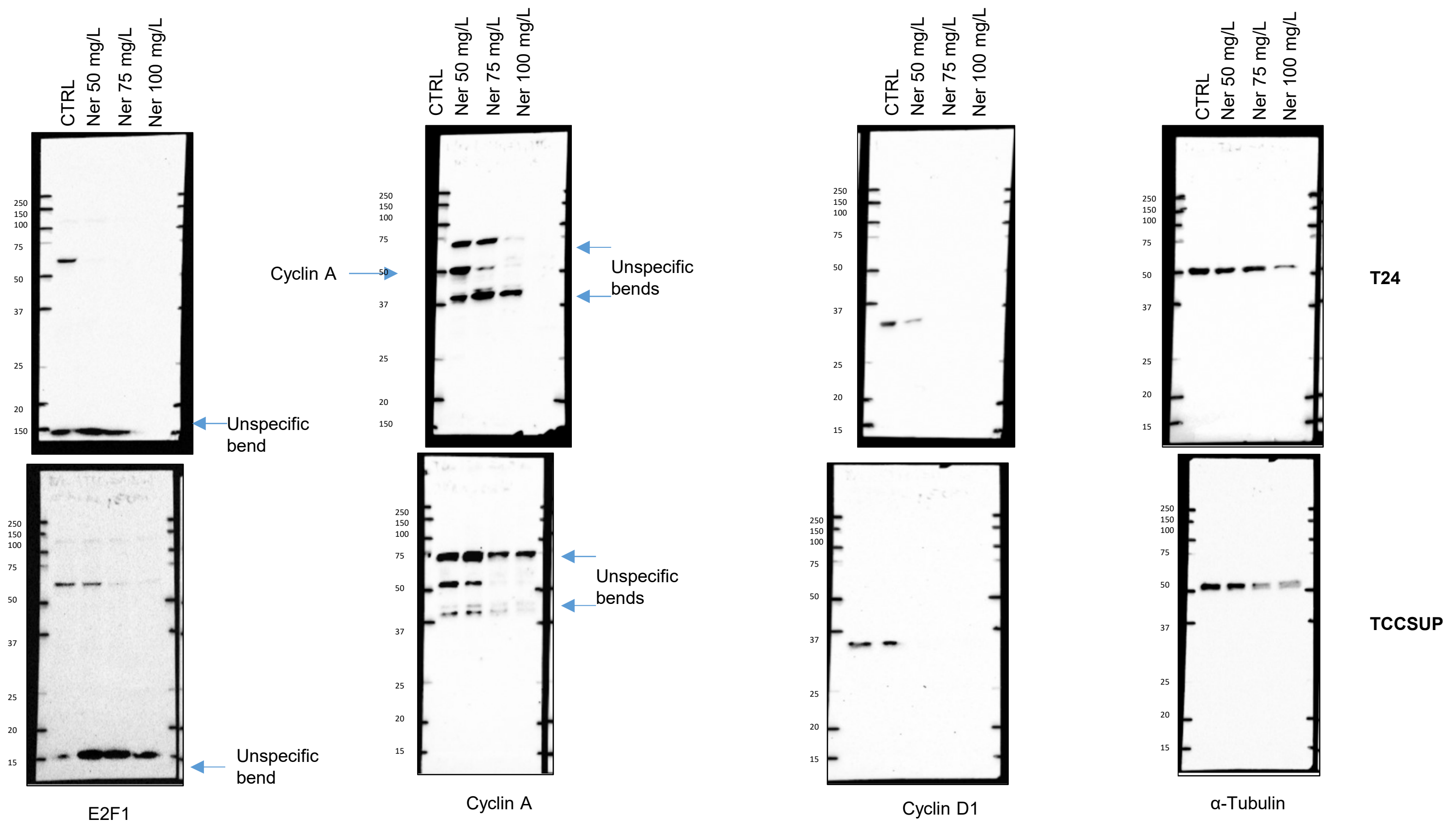
CTRL
Ner 50 mg/L
Ner 75 mg/L
Ner 100 mg/L
Cyclin A
Unspecific bend
Unspecific bends
T24
TCCSUP
E2F1
Cyclin A
Cyclin D1
α-Tubulin

Figure S1 D

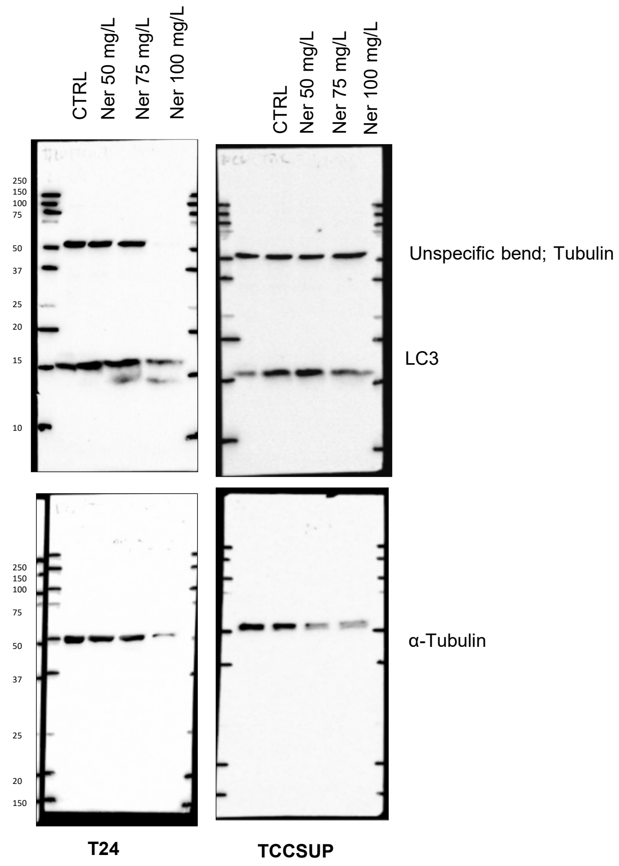

CTRL
Ner 50 mg/L
Ner 75 mg/L
Ner 100 mg/L
CTRL
Ner 50 mg/L
Ner 75 mg/L
Ner 100 mg/L
250
150
100
75
50
37
25
20
15
10
Unspecific bend; Tubulin
LC3
250
150
100
75
50
37
25
20
150
α-Tubulin
T24
TCCSUP

Figure S2 A

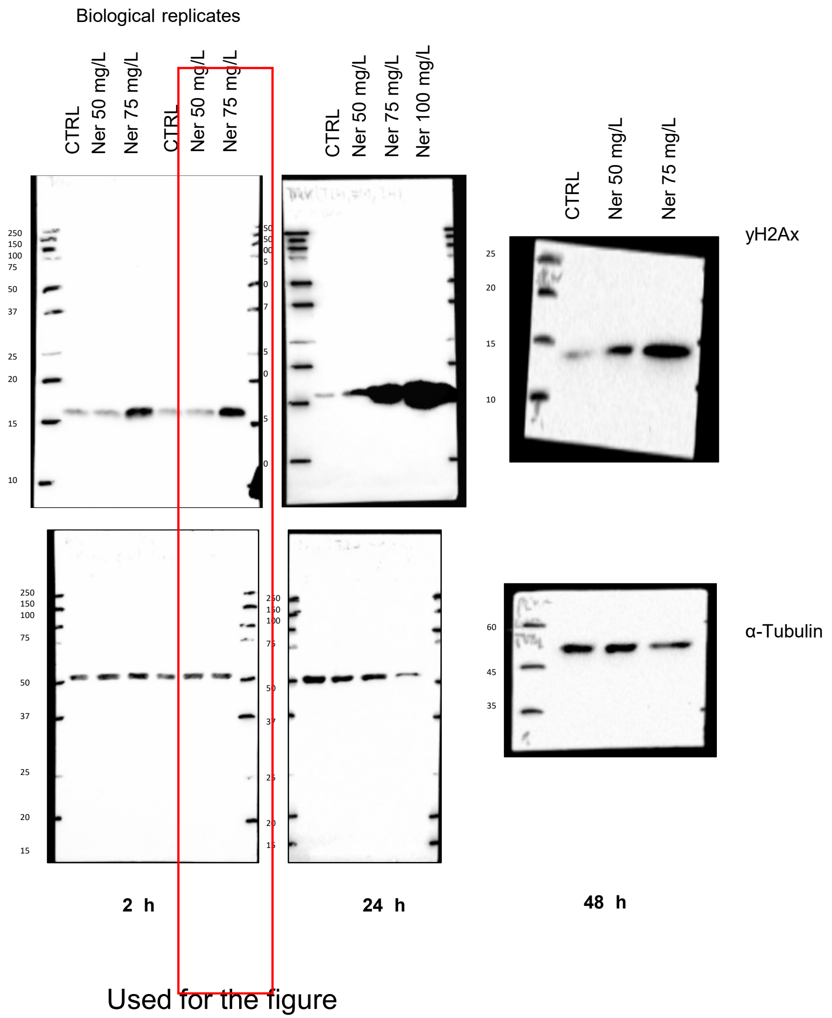

T24

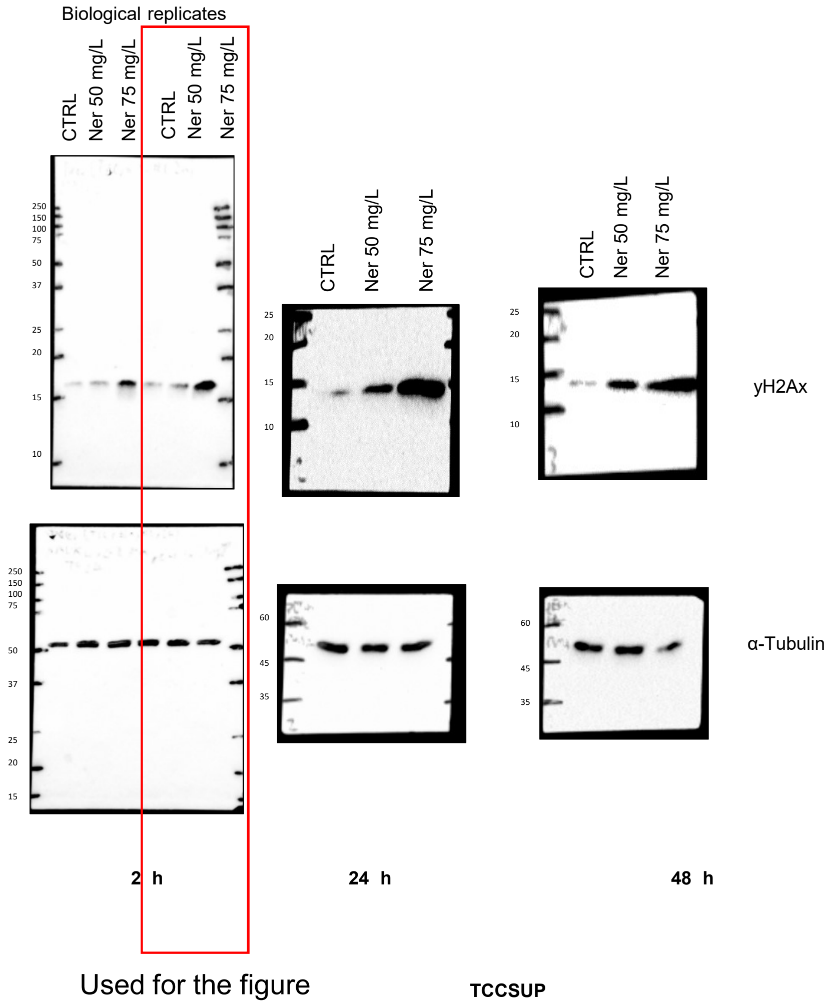

TCCSUP

Figure S2 C

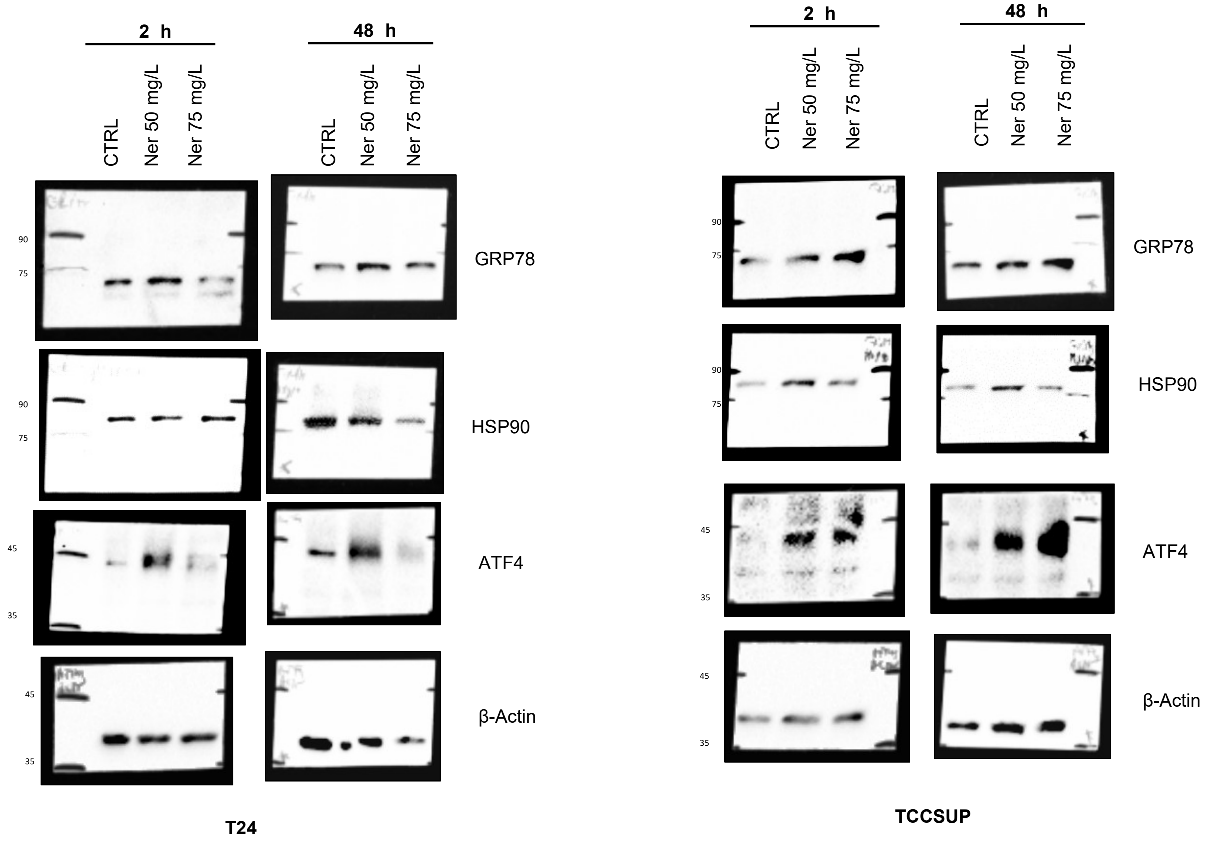
2 h
48 h
CTRL
Ner 50 mg/L
Ner 75 mg/L
CTRL
Ner 50 mg/L
Ner 75 mg/L
GRP78
HSP90
ATF4
β-Actin
T24
2 h
48 h
CTRL
Ner 50 mg/L
Ner 75 mg/L
CTRL
Ner 50 mg/L
Ner 75 mg/L
GRP78
HSP90
ATF4
β-Actin
TCCSUP

Figure S3 F

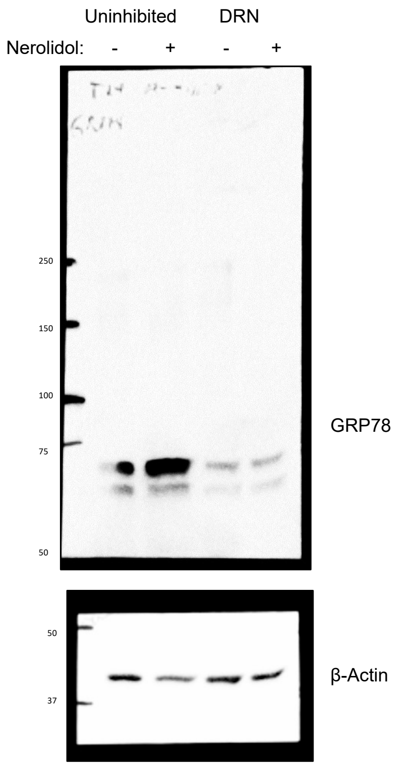
Uninhibited
DRN
Nerolidol:
-
+
-
+
250
150
100
75
50
GRP78
50
37
β-Actin

Figure S4 A

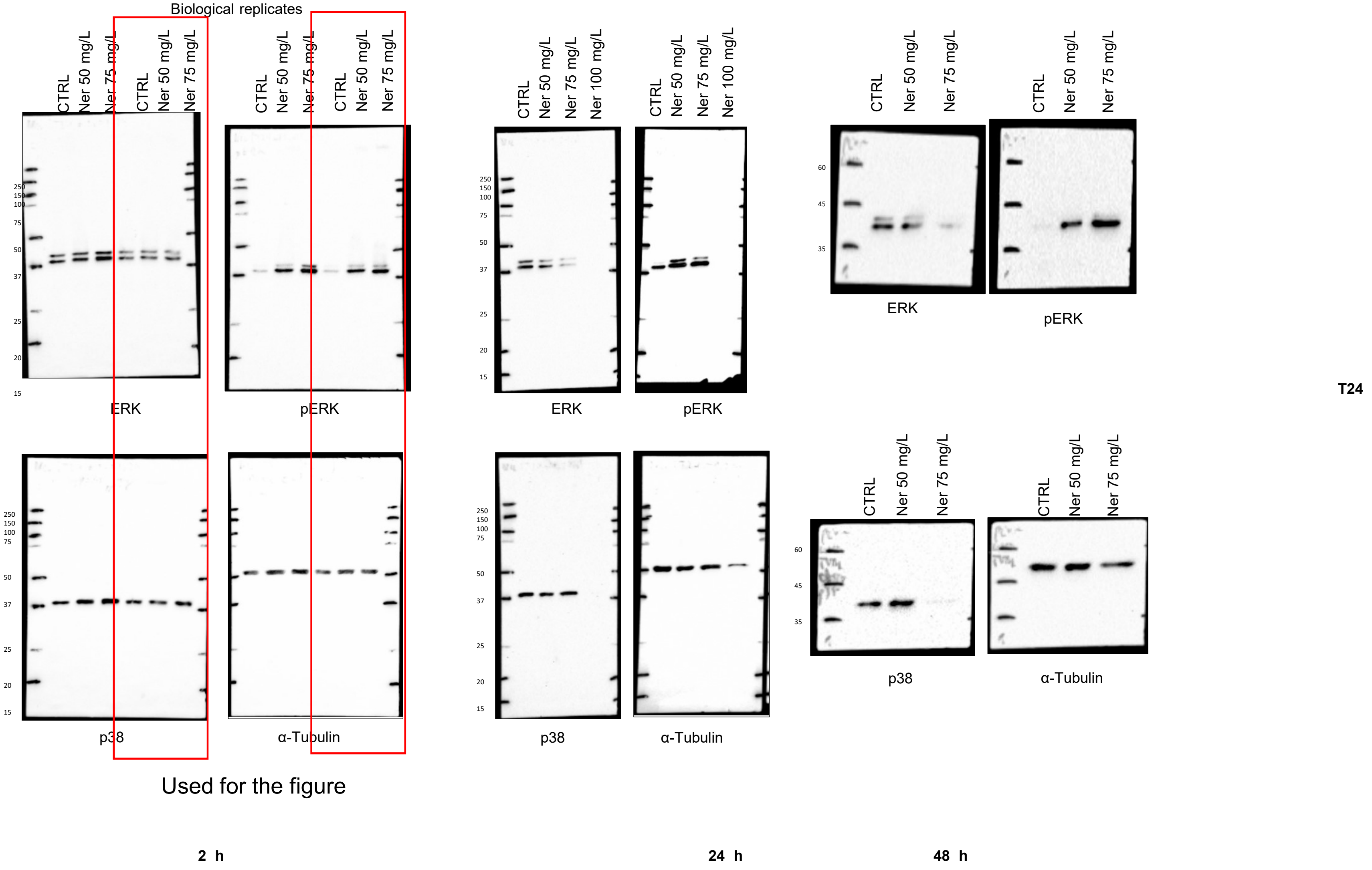

Biological replicates
CTRL
Ner 50 mg/L
Ner 75 mg/L
Ner 100 mg/L
250
150
100
75
60
50
45
37
35
25
20
15
ERK
pERK
p38
α-Tubulin
Used for the figure
T24
2 h
24 h
48 h

Figure S4 A

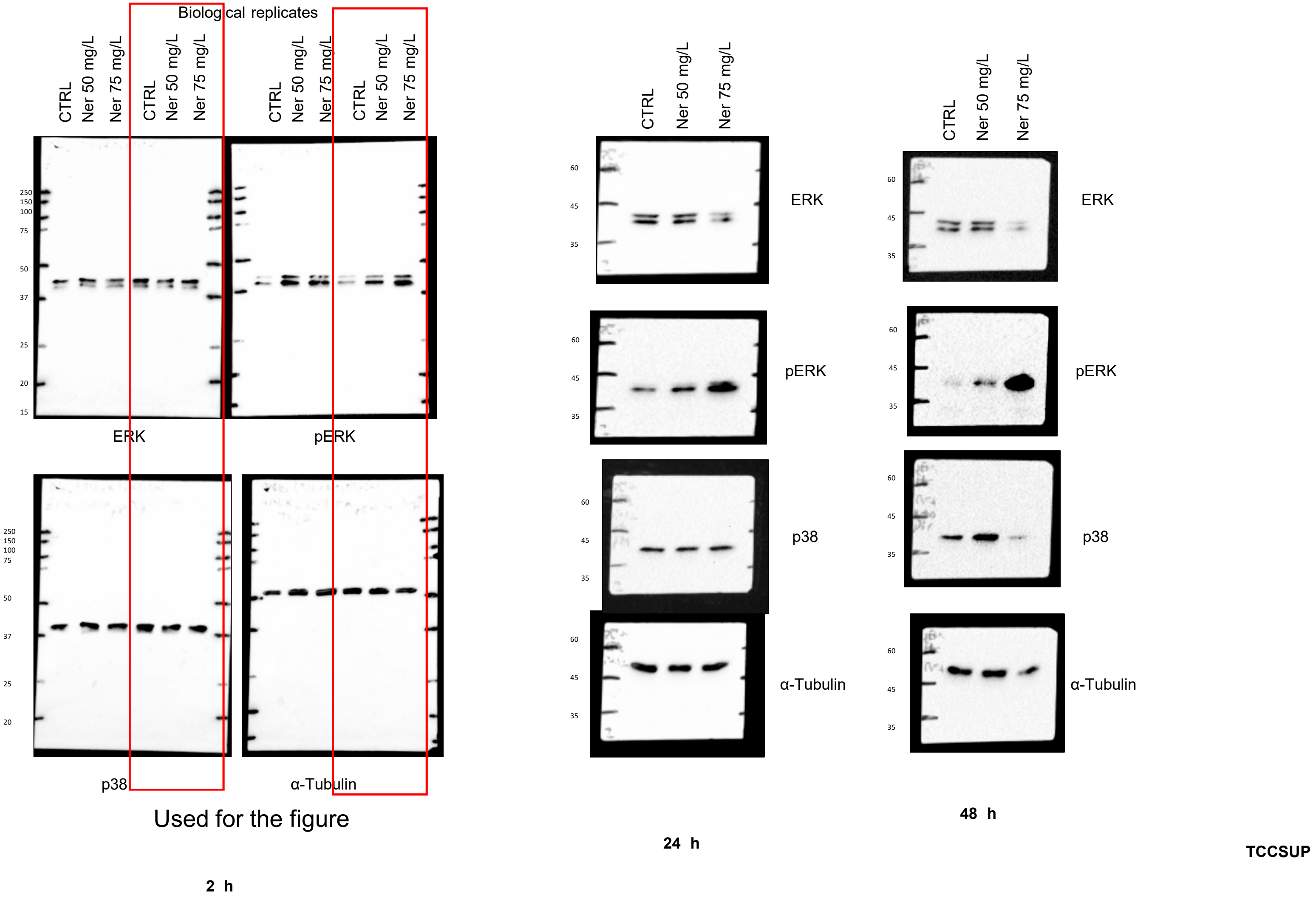

Biological replicates
CTRL
Ner 50 mg/L
Ner 75 mg/L
ERK
pERK
p38
α-Tubulin
Used for the figure
2 h
24 h
48 h
TCCSUP

Figure S4 E

Used for the figure

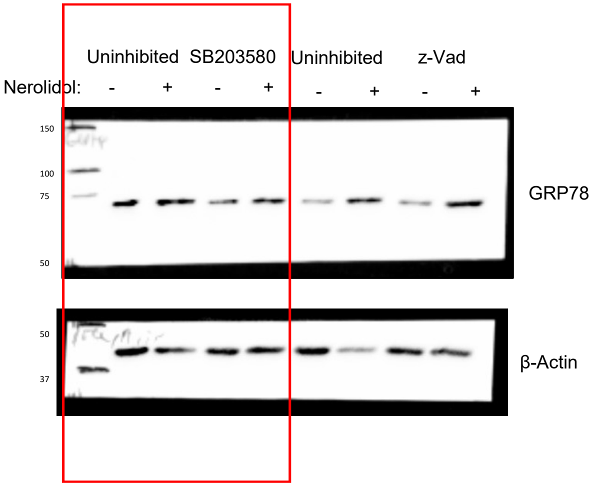

Figure S5 E

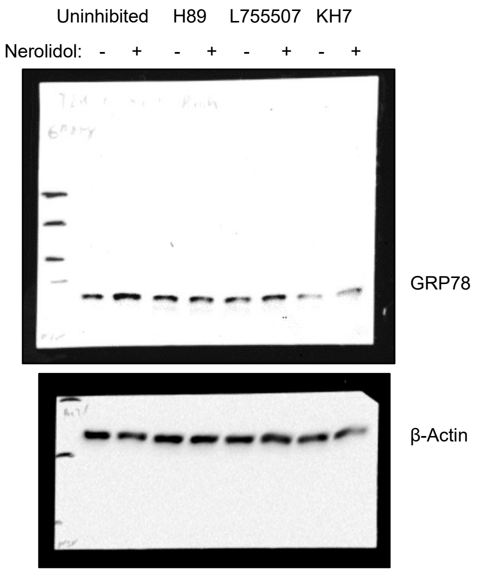

Figure S7 B

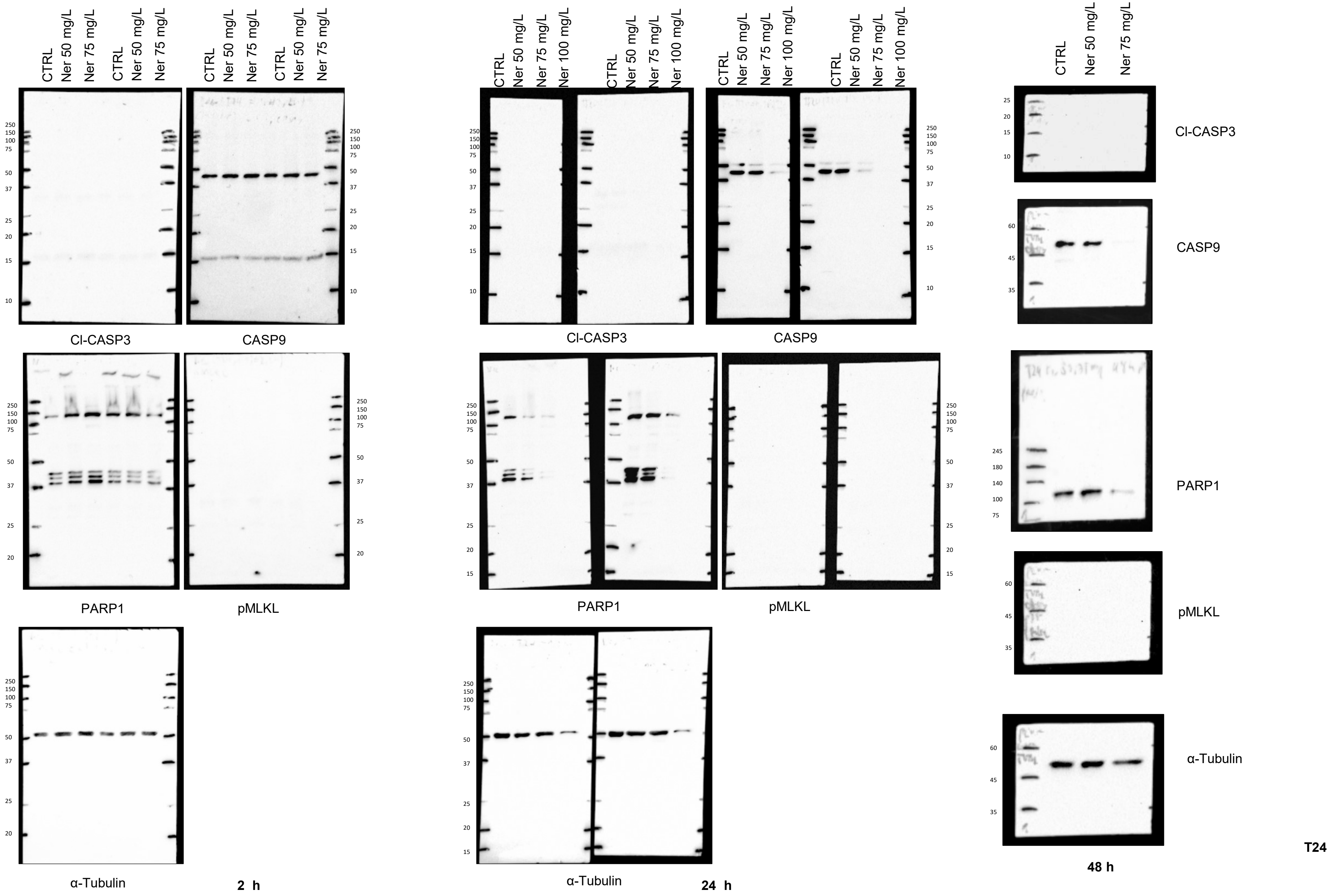

CTRL
Ner 50 mg/L
Ner 75 mg/L
Ner 100 mg/L
Cl-CASP3
CASP9
PARP1
pMLKL
α-Tubulin
2 h
24 h
48 h
T24

Figure S7 B

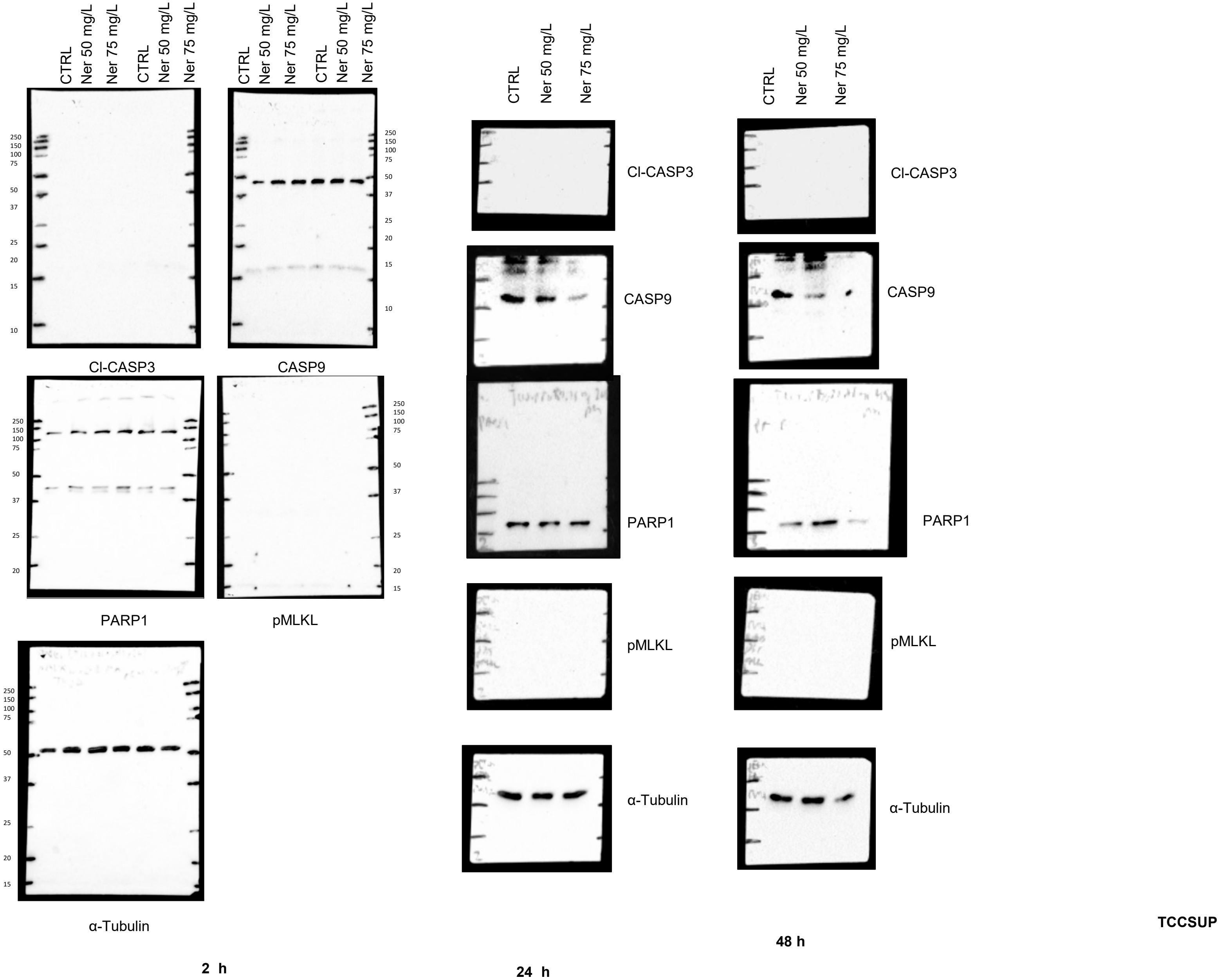

CTRL
Ner 50 mg/L
Ner 75 mg/L
CTRL
Ner 50 mg/L
Ner 75 mg/L
250
150
100
75
50
37
25
20
15
10
Cl-CASP3
CASP9
PARP1
pMLKL
α-Tubulin
2 h
24 h
48 h
TCCSUP

Figure S7 G

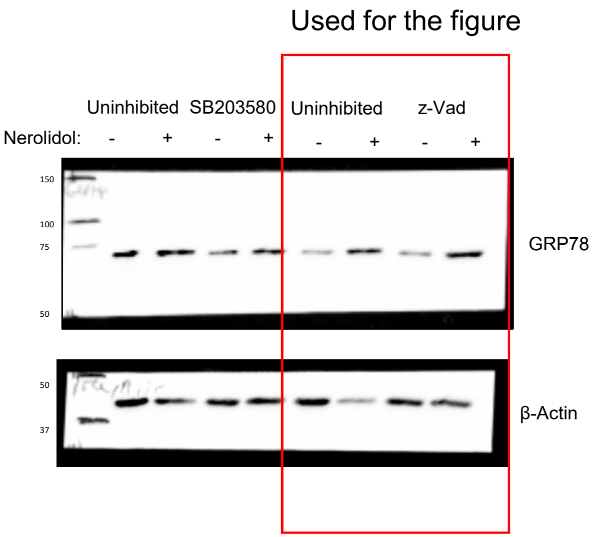

Used for the figure
Uninhibited SB203580 Uninhibited z-Vad
Nerolidol: - + - + - + - +
150
100
75
50
GRP78
50
37
β-Actin
