# Supplementary material for: Mechanism of cis-Nerolidol-Induced Bladder Carcinoma Cell Death
Source: Cancers (Basel). 2023 Feb 3;15(3):981. doi: 10.3390/cancers15030981 (PMC9913136; doi:10.3390/cancers15030981)
Supplement: Supplementary file 1 [file cancers-15-00981-s001.zip › Tables and Figures.pdf]

Supplementary figures and tables

Mechanism of *cis*-Nerolidol-Induced Bladder Carcinoma Cell Death

Mateo Glumac, Vedrana Čikeš Čulić, Ivana Marinović-Terzić, and Mila Radan

Figure S1. *cis*-nerolidol reduces cell viability through two cell death events with distinct cell morphologies

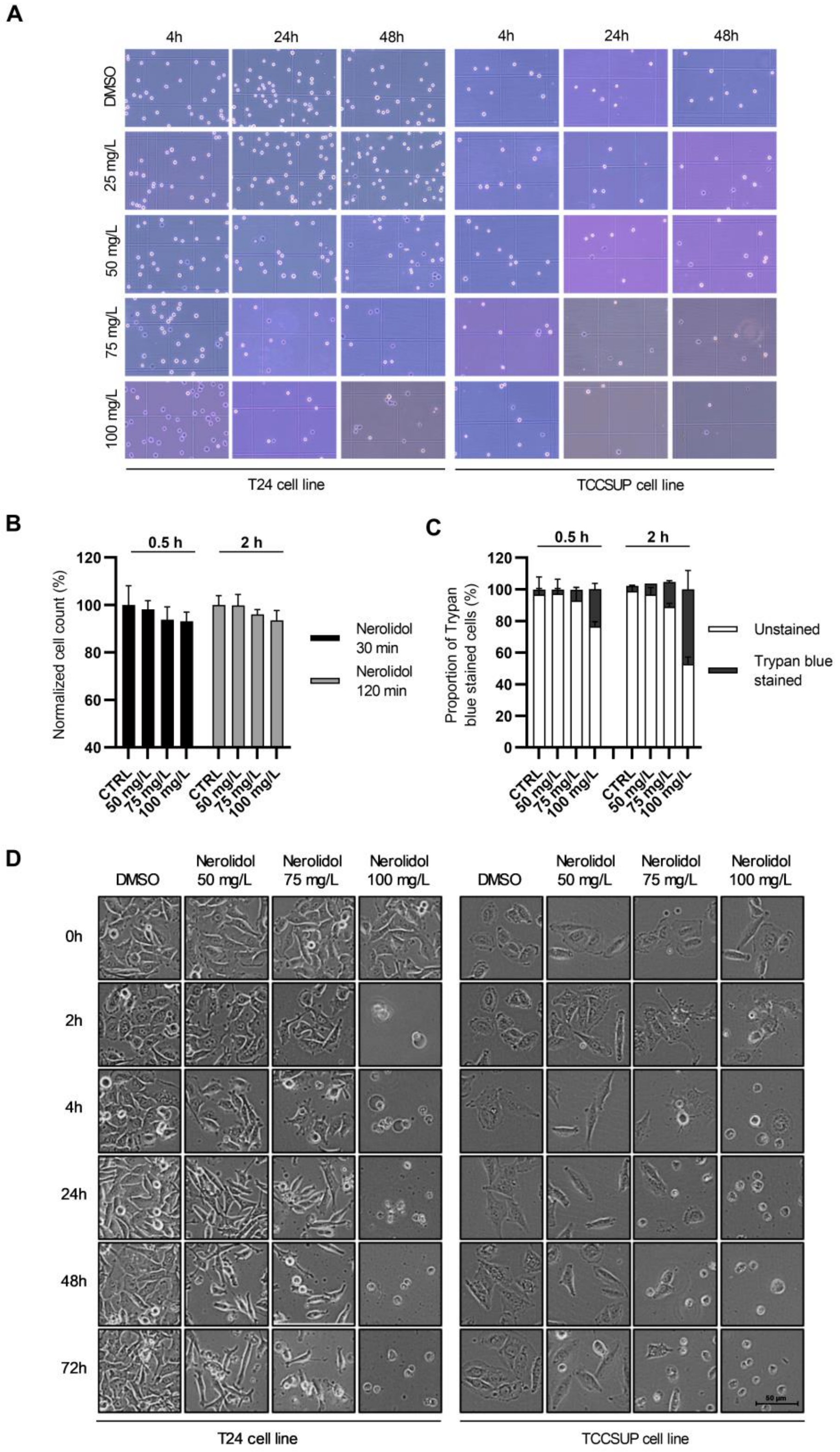

Figure S2. *cis*-nerolidol causes deregulation in ROS, ATP, and cAMP production

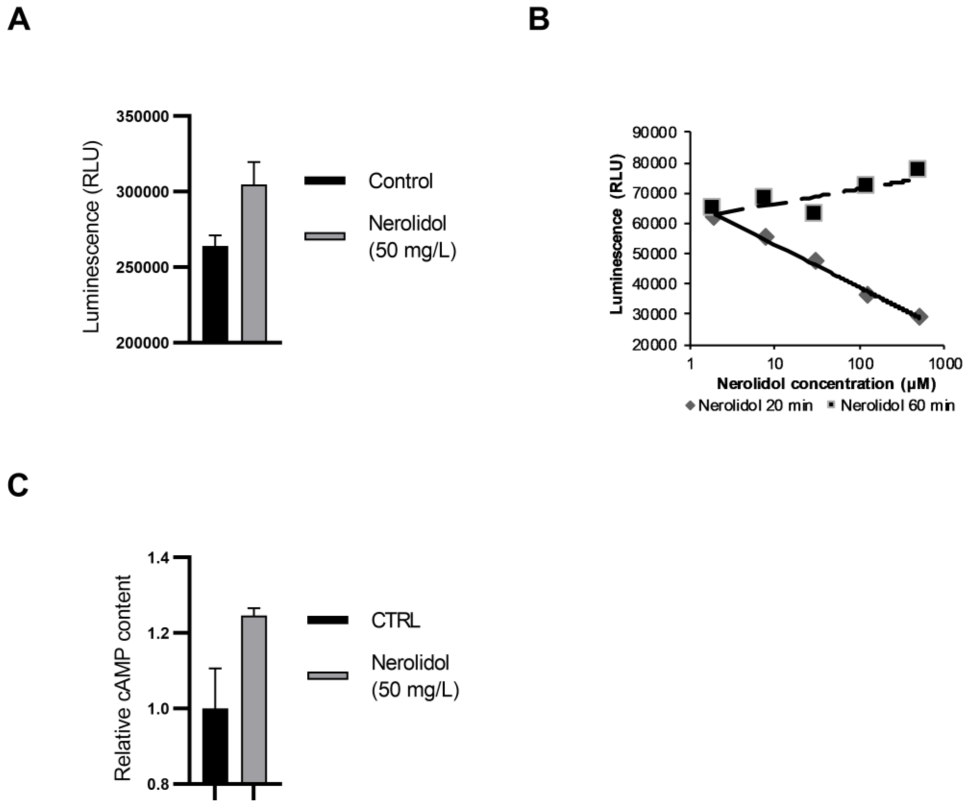

Figure S3. Short exposure to *cis*-nerolidol reduces cell proliferation and changes in cell morphology

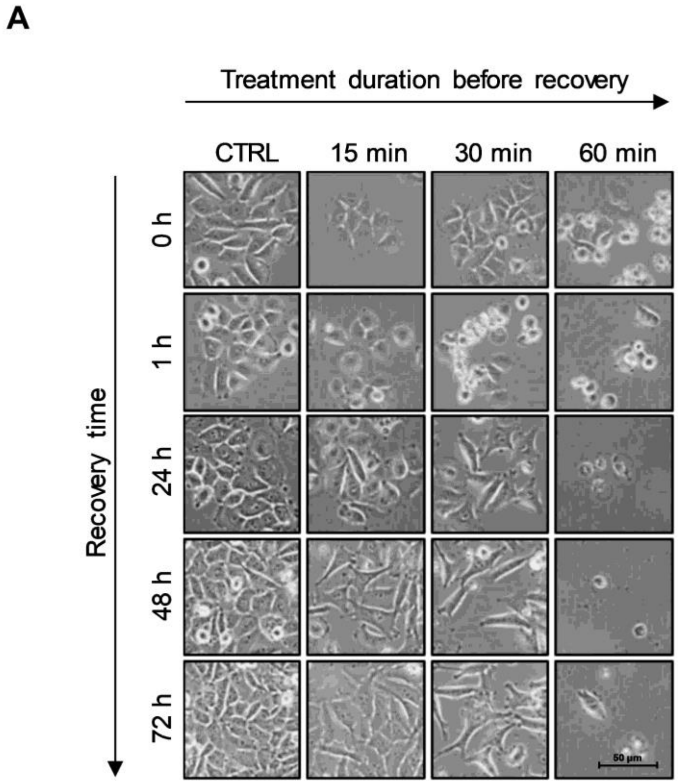

Figure S4. Signal transduction network involved in *cis*-nerolidol induced cell death

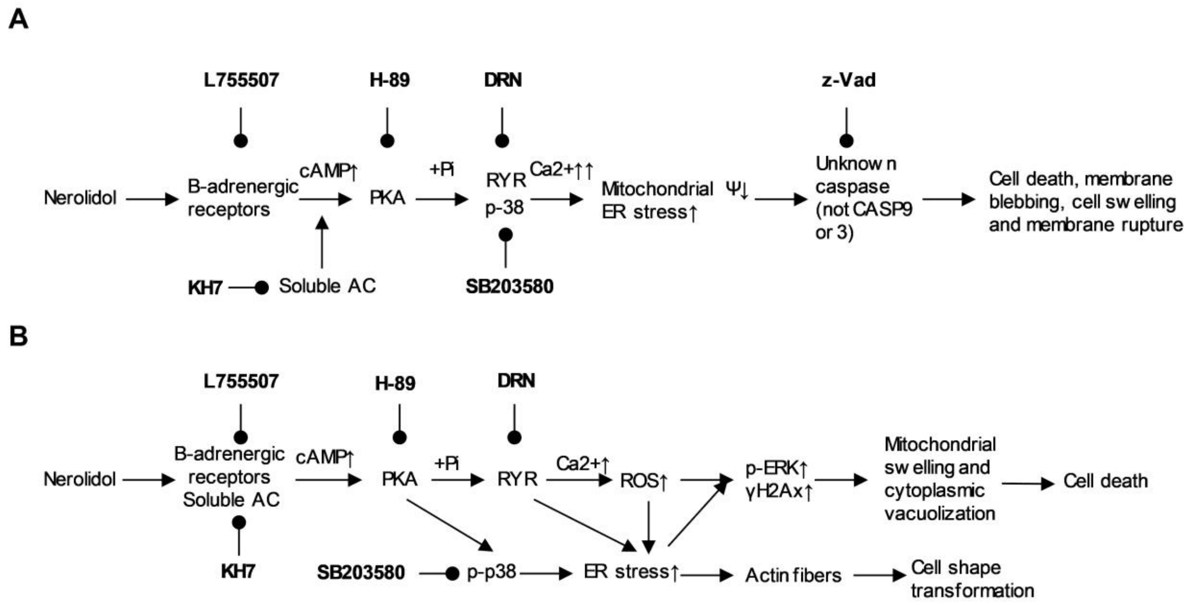

Table S1. IC_{50} values for reduction of cell count at different time points

**T24 cell line**	**4h**	**24h**	**48h**	**72h**
IC50 (mg/L)	>100	42.82 ± 3.88	37.06 ± 4.22	25.58 ± 2.82

**TCCSUP cell line**	**4h**	**24h**	**48h**	**72h**
IC50 (mg/L)	>100	71.92 ± 6.30	51.19± 7.52	46.59 ± 5.30
